# Wearable MEG data recorded during human stepping

**DOI:** 10.1016/j.dib.2025.111574

**Published:** 2025-04-25

**Authors:** Meaghan E. Spedden, George C. O’Neill, Timothy O. West, Tim M. Tierney, Stephanie Mellor, Nicholas A. Alexander, Robert Seymour, Jesper Lundbye-Jensen, Jens Bo Nielsen, Simon F. Farmer, Sven Bestmann, Gareth R. Barnes

**Affiliations:** a Department of Imaging Neuroscience, UCL Institute of Neurology, London WC1N 3AR, United Kingdom; b Department of Neuroscience, Physiology and Pharmacology, University College London, London, United Kingdom; c Department of Biomedical Engineering, Imperial College London, London, United Kingdom; d Department for Clinical and Movement Neuroscience, UCL Queen Square Institute of Neurology, University College London, WC1N 3BG, United Kingdom; e Department of Clinical Neurology, The National Hospital for Neurology and Neurosurgery, Queen Square London WC1N 3BG, United Kingdom; f Movement & Neuroscience, Department of Nutrition, Exercise and Sports, University of Copenhagen, Copenhagen, Denmark; g Department of Neuroscience, University of Copenhagen, Copenhagen, Denmark

**Keywords:** Optically-pumped magnetometers (OPM), Sensorimotor control, Naturalistic neuroimaging, Electromyography, Kinematics

## Abstract

Non-invasive spatiotemporal imaging of brain activity during large-scale, whole body movement is a significant methodological challenge for the field of movement neuroscience. Here, we present a dataset recorded using a new imaging modality – optically-pumped magnetoencephalography (OP-MEG) – to record brain activity during human stepping. Participants (n=3) performed a visually guided stepping task requiring precise foot placement while dual-axis and triaxial OP-MEG and leg muscle activity (electromyography, EMG) were recorded. The dataset also includes a structural MRI for each participant and foot kinematics. This multimodal dataset offers a resource for methodological development and testing for OPM data (e.g., movement-related interference rejection), within-subject analyses, and exploratory analyses to generate hypotheses for further work on the neural control of human stepping.

Specifications Table

**Table:** 

Subject	Biology
Specific subject area	Wearable MEG
Type of data	Raw
Data collection	Magnetoencephalography (MEG) data were collected using dual- and triaxial QuSpin manufactured optically-pumped magnetometers (OPM) in a magnetically shielded room. Sensors were positioned in custom built 3D printed rigid scanner casts constructed from each participant’s structural MRI. Electromyography (EMG) was recorded from the right anterior tibial muscle (TA) and foot kinematics were recorded using OptiTrack infrared cameras and retroreflective markers.
Data source location	Functional Imaging Laboratory, University College London, United Kingdom
Data accessibility	Repository name: Mendeley Data Data identification number: 10.17632/p3dfxmky46.4 Direct URL to data: https://data.mendeley.com/datasets/p3dfxmky46/4
Related research article	

## 1 Value of the Data

• This dataset is the first publicly available ambulatory OP-MEG dataset, enabling researchers to explore brain activity during whole-body movement and assess the potential of OP-MEG for movement neuroscience.
• It includes OP-MEG recordings with individual structural MRIs, supporting high-fidelity source reconstruction and the investigation of step-related cortical dynamics.
• The dataset contains lower-leg EMG recordings, facilitating analyses of cortico-muscular interactions using methods like cortico-muscular coherence.
• Task performance metrics and foot kinematics allow researchers to link neural activity with movement characteristics, supporting within-subject analyses of behaviour and motor control.
• Rich multimodal data make this dataset valuable for evaluating movement-related interference rejection methods and serve as a foundation for future large-scale studies.

## 2 Background

Walking is a fundamental behavior integral to all humans and animals that enables us to move around our environment. Similar to other animals, humans have a spinal cord network, known as a central pattern generator, that is responsible for generating the fundamental walking pattern [1,2]. However, in humans, the function of this network is more reliant on input from the brain and has been adapted to meet the specific demands of bipedal walking [3].

Studying brain activity non-invasively during large-scale movements is challenging due to the complex, multi-limb, multi-joint actions, and whole-body translation involved. A new approach that holds significant potential for progress in this field is optically-pumped magnetoencephalography (OP-MEG) [4,5]. OPMs are magnetic sensors that do not require cryogenic cooling, which means that they can be positioned within a few millimeters of the scalp in wearable arrays, offering a flexibility similar to EEG [6,7]. Magnetic field-based imaging also offers the advantage of improved spatial resolution [8,9] and lower sensitivity to muscle artifacts [10] relative to EEG.

Here, we present a multimodal dataset [11] including OP-MEG, structural MRI, foot kinematics, and leg muscle activity from three participants performing a visually guided stepping task. A validation analysis of this data is published as a preprint [12].

## 3 Data Description

Each dataset comprises OP-MEG data; electromyography (EMG) data; structural MRI; and kinematics/task performance information. All data are de-identified and available on Mendeley Data with DOI:10.17632/p3dfxmky46.4, and data are predominantly organised according to Brain Imaging Data Structure (BIDS). BIDS is a standard for organizing and describing neuroimaging and behavioural data to facilitate sharing, analysis, and reproducibility. BIDS provides a consistent framework for naming files, defining metadata, and structuring datasets, making it easier for researchers to collaborate and integrate data from different studies. Note that BIDS does not yet officially support OPMs, so we have used traditional MEG conventions.

The dataset is organized as follows:

• The main folder includes a **data folder for each participant**, a participants.tsv file listing participant IDs and ages, and a README.txt with synchronization trigger details.
• Each participant's folder contains a **session folder (ses-001)**, following the BIDS structure.
• Within the session folder, data is categorized into subfolders:
  ○ **anat** – Anatomical (structural MRI) data
  ○ **beh** – Behavioral (task performance) data
  ○ **emg** – Electromyography data
  ○ **meg** – Magnetoencephalography data

Table 1 provides an overview of the files within these folders and their contents. Note that we have published the raw data, which is not synchronized, and that the user must use trigger information to align OPM EMG and kinematic data (see README.txt).

**Table 1:** Overview of file name organisation and content.

Filename template	Contains	Notes
sub-OP00XXX_ses-001_task-**stepping**_run-XXX_meg.bin sub-OP00XXX_ses-001_task-**noise**_run-XXX_meg.bin	Raw OPM data	Each dataset also includes a noise (empty room) recording (task-noise); see ‘Electrophysiological recordings’ for details of noise recordings.
sub-OP00XXX_ses-001_task-stepping_run-XXX_meg.json	Meta data for OPM	
sub-OP00XXX_ses-001_task-stepping_run-XXX_channels.tsv	OPM channel name and type	For subject 00159 channel 39’s name is formatted differently because we did not record from this channel. This does not affect processing but needs to remain in tsv file for metadata compliance.
sub-OP00XXX_ses-001_task-stepping_run-XXX_positions.tsv	OPM sensor positions and orientations	Channel name, position, and orientation. OPM sensors in same coordinate space as MRI
sub-OP00XXX_ses-001_task-stepping_run-XXX_emg.tsv	EMG data	EMG signal from right anterior tibial muscle
sub-OP00XXX_ses-001_task-stepping_run-XXX_emg.json	Meta data for EMG	
OP00XXX-defaced.nii	Structural MRI	
sub-XXX_ses-001_task-stepping_recording-kinematics_beh.tsv	Kinematics and task information	3D foot (rigid body) position; 2D foot and target position; trigger timing

## 4 Experimental Design, Materials and Methods

### 4.1 Participants

Three healthy participants (Age 55, 33, and 30; all male) participated in this study. Written, informed consent was obtained prior to participation and the experimental protocol was approved by the University College London Research Ethics Committee.

### 4.2 Stepping Task and Kinematics

Participants performed a visually guided stepping task while we recorded OP-MEG and concurrent EMG from the tibialis anterior (TA) muscle. The stepping task was an adapted version of a visually guided walking paradigm used in previous work [13,14] which demonstrated the presence of cortico-muscular and cortico-cortical coupling during stepping.

**Fig. 1:**
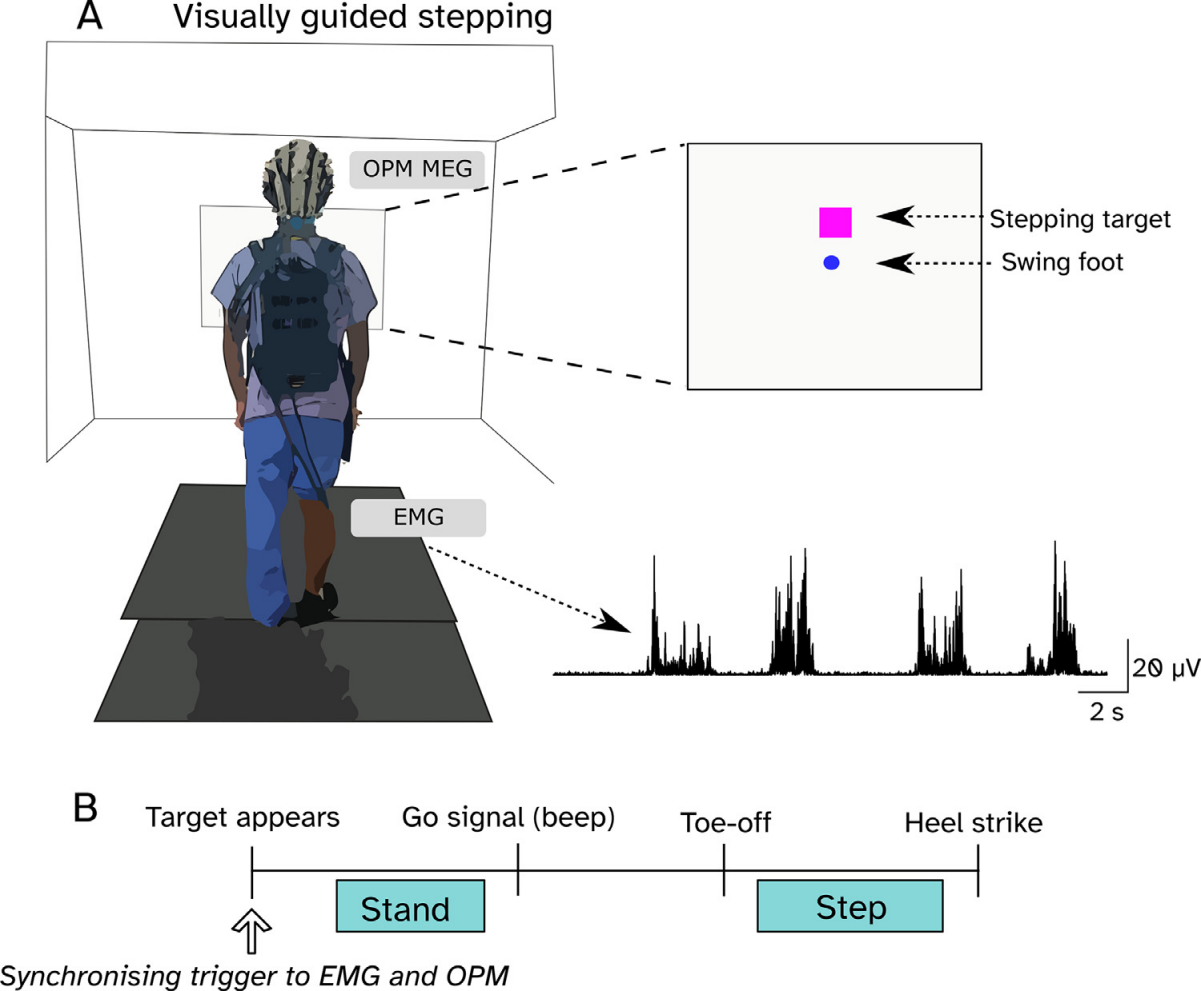
Experimental setup. In the visually guided stepping task (A) participants took single steps forwards aiming to hit a virtual target (magenta square) with a blue circle reflecting the position of their right foot. OP-MEG and EMG from the right tibialis anterior (TA) were recorded.

The task required participants to take single steps using their right leg, aiming for virtual stepping targets. The stepping target and real-time position of the stepping leg were projected on a screen, represented as a magenta square and blue circle, respectively (Fig. 1A). The task involved using the real-time visual feedback of the stepping leg position relative to the stepping target to adjust step length and hit the target. Stepping leg position was tracked using a set of six infrared cameras at 120 Hz (Optitrack, Flex 3, Natural Point, Inc.) to record the position of retro-reflective markers placed on the stepping foot. The markers were used to construct a rigid body of the foot, and its coordinates were streamed using the Motive (Natural Point, Inc.) NatNet SDK to MATLAB, where a custom script ran the stimulus presentation.

Prior to the start of the experiment, the virtual target distance was adjusted to represent a comfortable step length for the participant (preferred step length). During recordings, target distance (step length in the anterior–posterior direction) was drawn randomly from 3 possible values: preferred step length, preferred length +5 cm and preferred length -5 cm. Participants began each trial standing quietly and took a step forward with the right leg aiming to hit the magenta square (target) with the blue circle, which moved with the right foot. The trial started when the target was projected on the screen, and participants were instructed to initiate the step when they heard a beep serving as the go signal. The trial was completed when the left foot was placed next to the right foot, and the participant then returned to the starting position, which was marked as an open circle on the screen. Trial duration was ∼10 seconds, and 5-6 blocks of 30 steps each (5 for participants OP00054 and OP00061; 6 for participant OP00159) were recorded.

The custom MATLAB script sent synchronizing triggers to OPM and EMG acquisition systems upon target appearance for each trial. The script also wrote rigid body kinematics, trigger timing, target position, 2D foot position projection (blue circle) to a text file. The stimulus presentation code is available on GitHub (https://github.com/meaghanspedden/stepping_opm_data).

### 4.3 Electrophysiological Recordings

#### 4.3.1 Optically Pumped Magnetometer-Based Magnetoencephalography (OP-MEG)

**Fig. 2:**
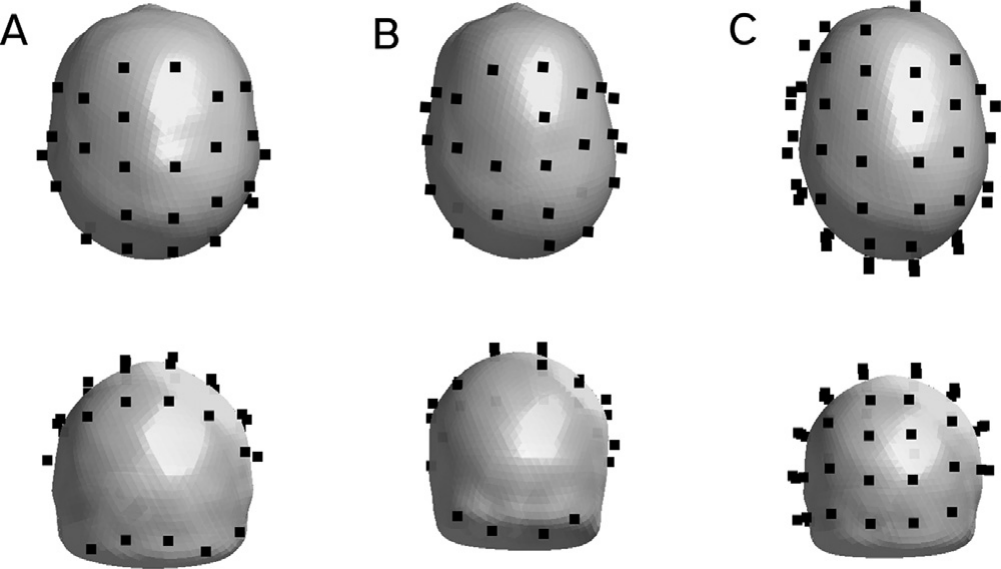
OPM sensor coverage. Coverage is shown for each participant (A-C, respectively). Sensors are depicted as black squares on the scalp mesh derived from each participant’s MRI. First row: superior view. Second row: posterior view.

The experiments were performed in an MSR (Magnetic Shields, Ltd., Staplehurst, UK; internal dimensions 3 × 4 × 2.2 m). The room was degaussed prior to the start of the experiment. Dual axis and triaxial OP-MEG sensors (QuSpin Inc., Louisville, CO, USA) were positioned in sockets in a rigid scanner-cast constructed from each participant’s structural MRI (Participant 1: 30 dual-axis sensors; participant 2: 27 dual-axis sensors; and participant 3: 47 triaxial sensors. See Fig. 2 for sensor layouts). This ensures accurate co-registration, maximal signal for any head size, and the rigidity minimizes sensor and cable movement relative to the head. For participants 1 and 2, OP-MEG data was acquired using a National Instruments acquisition system and a custom LABVIEW program with a sampling frequency of 6000 Hz. An antialiasing 500 Hz low-pass filter (60th order FIR filter combined with a Kaiser window) was applied before data were down-sampled offline to 2 kHz. Sensors were operated in a mode with a dynamic range of ± 4.5 nT. For participant 3, we used the Neuro-1 acquisition system (QuSpin Inc., Louisville, CO, USA) consisting of exclusively tri-axial sensors (in open loop mode) with a sampling frequency of 1500 Hz. Both dual-axis and triaxial sensors have an intrinsic bandwidth of 0–135 Hz (due to the properties of the vapour cell). In the Neuro-1 acquisition system the manufacturer has additionally implemented a high order digital low pass FIR filter at 150 Hz.

Before each experiment, empty room recordings (*task-noise*) were performed with the sensors positioned in the scanner cast on a table in the centre of the shielded room. These recordings can be used to generate e.g. a power spectrum reflecting frequency content of environmental noise in the shielded room.

#### 4.3.2 Electromyography (EMG)

EMG was recorded from the right tibialis anterior (TA) muscle. Two surface electrodes (2 cm diameter, Natus Neurology, Inc.) were placed over the centre of the muscle (ca. 2 cm between electrodes) and a ground electrode was positioned on the right lateral malleolus. To prevent EMG data collection from interfering with OPM signals, we passed EMG cables through waveguides so that EMG signals were amplified, filtered, and digitized outside the MSR (Amplification: x 1000; hardware filtering 3 to 100 Hz and 50 Hz notch D-360 amplifier; digitization at 1000 Hz, and 1401 data acquisition unit, Cambridge Electronic Design, UK). Signals were recorded using Spike2 software (v10.05).

Software packages suitable for analysing the data include SPM [15], Fieldtrip [16], and MNE-Python [17]. We provide example MATLAB code on GitHub (https://github.com/meaghanspedden/stepping_opm_data) to demonstrate how this dataset can be used for source imaging of movement-related beta power. This analysis is also presented in our corresponding preprint [12]. Another demonstrative case of how similar data can be analysed and used for research can be found in [18] where we show that cortical representations of limbs can be imaged during whole body movement using OPMs.

## Limitations

A limitation of the dataset is that it contains data from three participants. While not suitable for group-level analysis, it is ideal for rich and comprehensive within-participant analyses [19] (due to its multimodal nature) and comparisons with EEG data collected using the same paradigm [13]. It also serves as a tool for developing and refining movement-related interference rejection methods. Spatial and spatiotemporal filtering techniques such as homogeneous field correction [20] and adaptive multipole modelling [21] are already highly effective but continue to evolve. The code for these methods is freely available in the Statistical Parametric Mapping MATLAB toolbox (https://github.com/spm) providing a useful point of departure. Ongoing advancements in these types of methods hold great potential for further enhancing data quality by mitigating movement-related noise and other environmental and physiological interference in OPM data.

Another potential limitation is that these data were recorded across two different OPM systems. A comparison of the electronics of the two systems can be found in ref [22]. In short, the Neuro-1 system has more channels, which can improve spatial filtering and denoising [21], and its electronics produce less noise, providing more accurate timing, especially for frequencies above 100 Hz. The noise floor also increases when one moves from dual-axis (15fT/sqrt(Hz) to tri-axial sensors (23fT/sqrt(Hz)). Despite these differences, existing work suggests that the two acquisition systems produce comparable results. When measuring task-induced beta-band, gamma-band, and evoked responses, results from both systems showed striking temporal and spatial consistency [22]. Our validation analysis of this dataset also supports congruent spatial results between the two types of acquisition systems [11].

## Ethics Statement

Written, informed consent was obtained prior to participation and the experimental protocol was approved by the University College London Research Ethics Committee (15889.001). Experiments were conducted in accordance with the Declaration of Helsinki.

## CRediT authorship contribution statement

**Meaghan E. Spedden:** Conceptualization, Methodology, Software, Writing – original draft, Writing – review & editing. **George C. O’Neill:** Software, Writing – review & editing. **Timothy O. West:** Software, Writing – review & editing. **Tim M. Tierney:** Software, Writing – review & editing. **Stephanie Mellor:** Software, Writing – review & editing. **Nicholas A. Alexander:** Software, Writing – review & editing. **Robert Seymour:** Software, Writing – review & editing. **Jesper Lundbye-Jensen:** Conceptualization, Methodology, Writing – review & editing. **Jens Bo Nielsen:** Conceptualization, Methodology, Writing – review & editing. **Simon F. Farmer:** Conceptualization, Methodology, Writing – review & editing. **Sven Bestmann:** Software, Writing – review & editing. **Gareth R. Barnes:** Conceptualization, Methodology, Software, Writing – review & editing.

## Data Availability

Mendeley DataWearable MEG recorded during human stepping (Original data).
